# Supplementary material for: Skin, but not gut, microbial communities vary with social density in Antarctic fur seals
Source: Front Microbiol. 2025 Jul 2;16:1603500. doi: 10.3389/fmicb.2025.1603500 (PMC12265080; doi:10.3389/fmicb.2025.1603500)
Supplement: Supplementary file 1 [file Data_Sheet_1.pdf]

Supplementary Figures and Tables

Manuscript title: Skin, but not gut, microbial communities vary with social density in Antarctic fur seals

Petroula Botsidou

- Supplementary figures
    - **Figure S1.**
    - **Figure S2.**
    - **Figure S3.**
- Supplementary tables
    - **Table S1.**
    - **Table S2.**
    - **Table S3.**
    - **Table S4.**
    - **Table S5.**
    - **Table S6.**
    - **Table S7.**
    - **Table S8.**
    - **Table S9.**
    - **Table S10.**

Supplementary figures

Figure S1.

Rarefaction curves for skin (A) and gut (B) samples showing the number of observed ASVs in relation to the sequencing depth of every sample.

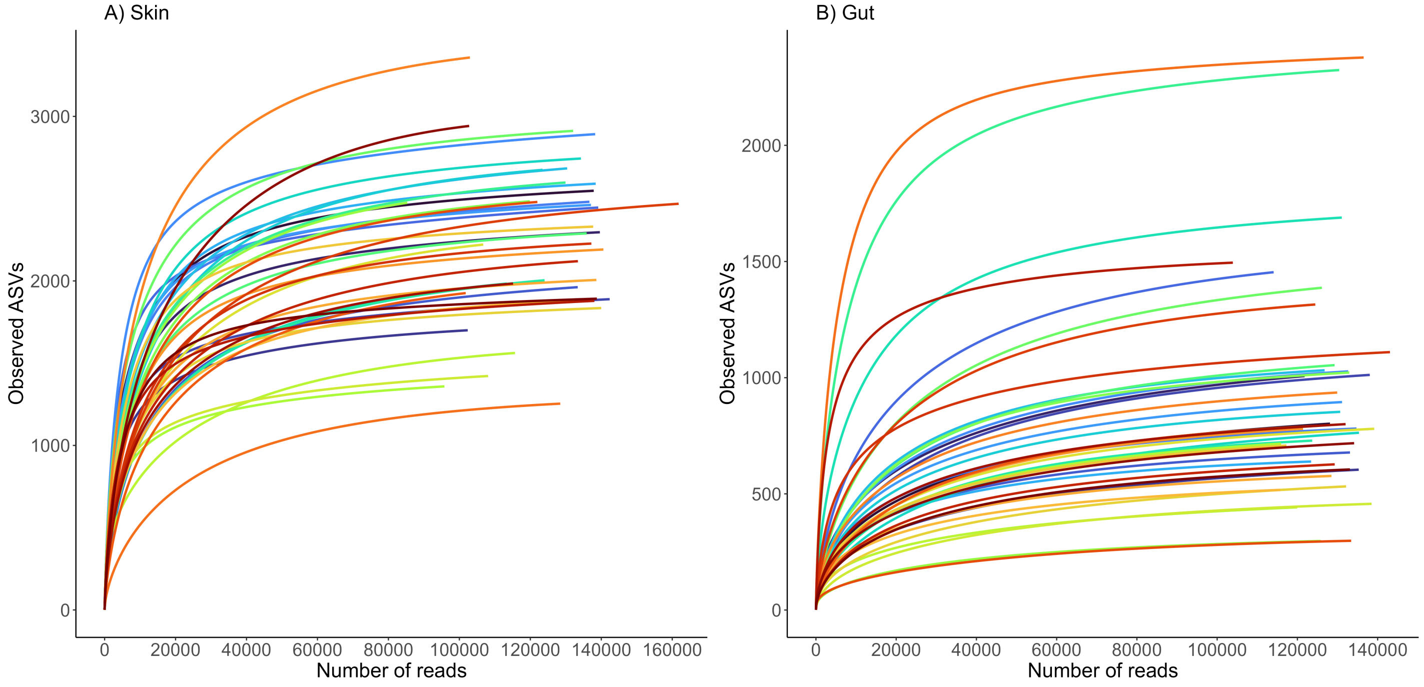

Figure S2.

Distribution of Shannon diversity index for skin and gut samples presented with boxplots.

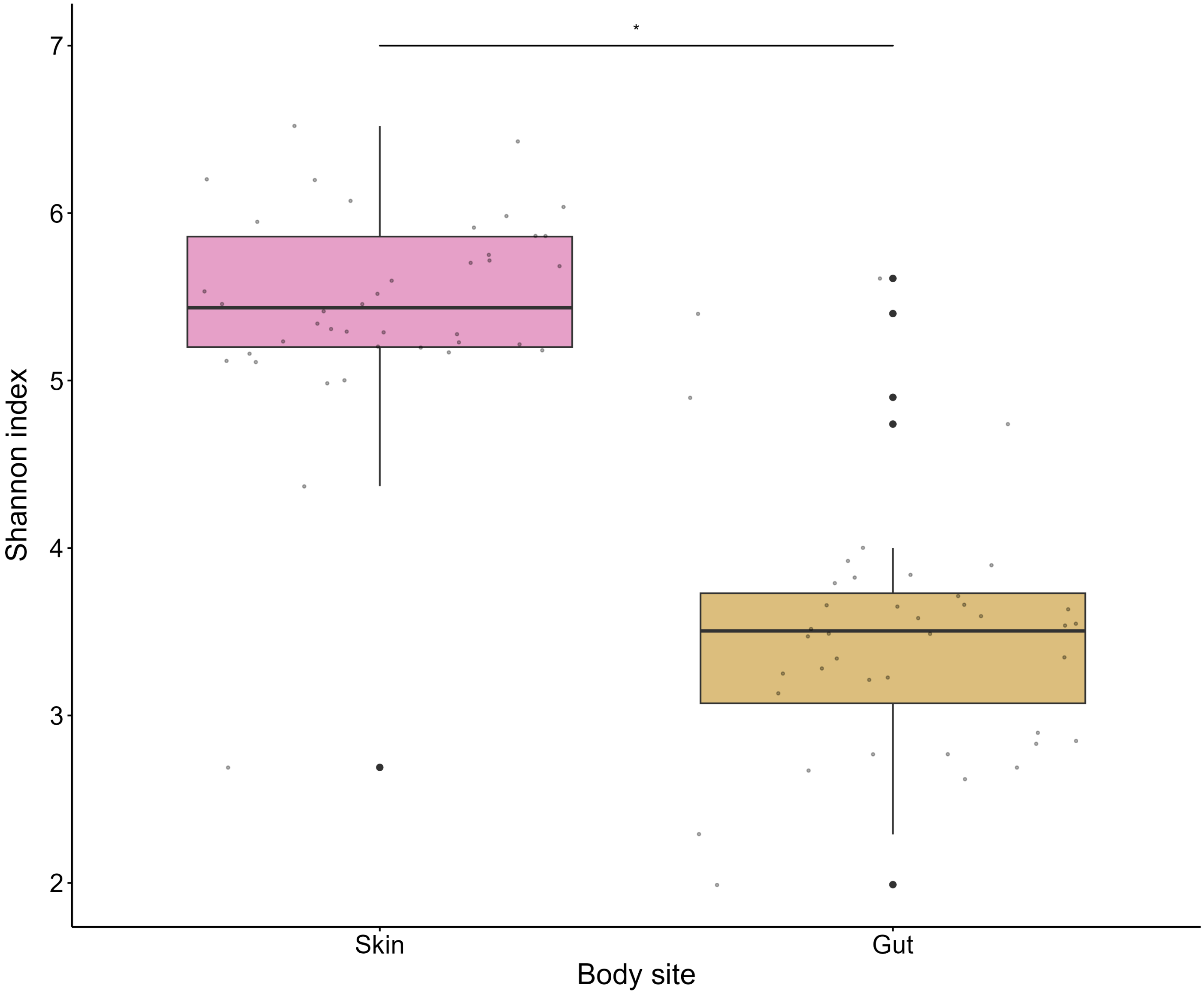

Figure S3.

Shannon diversity index for skin samples including outlier (pup H11).

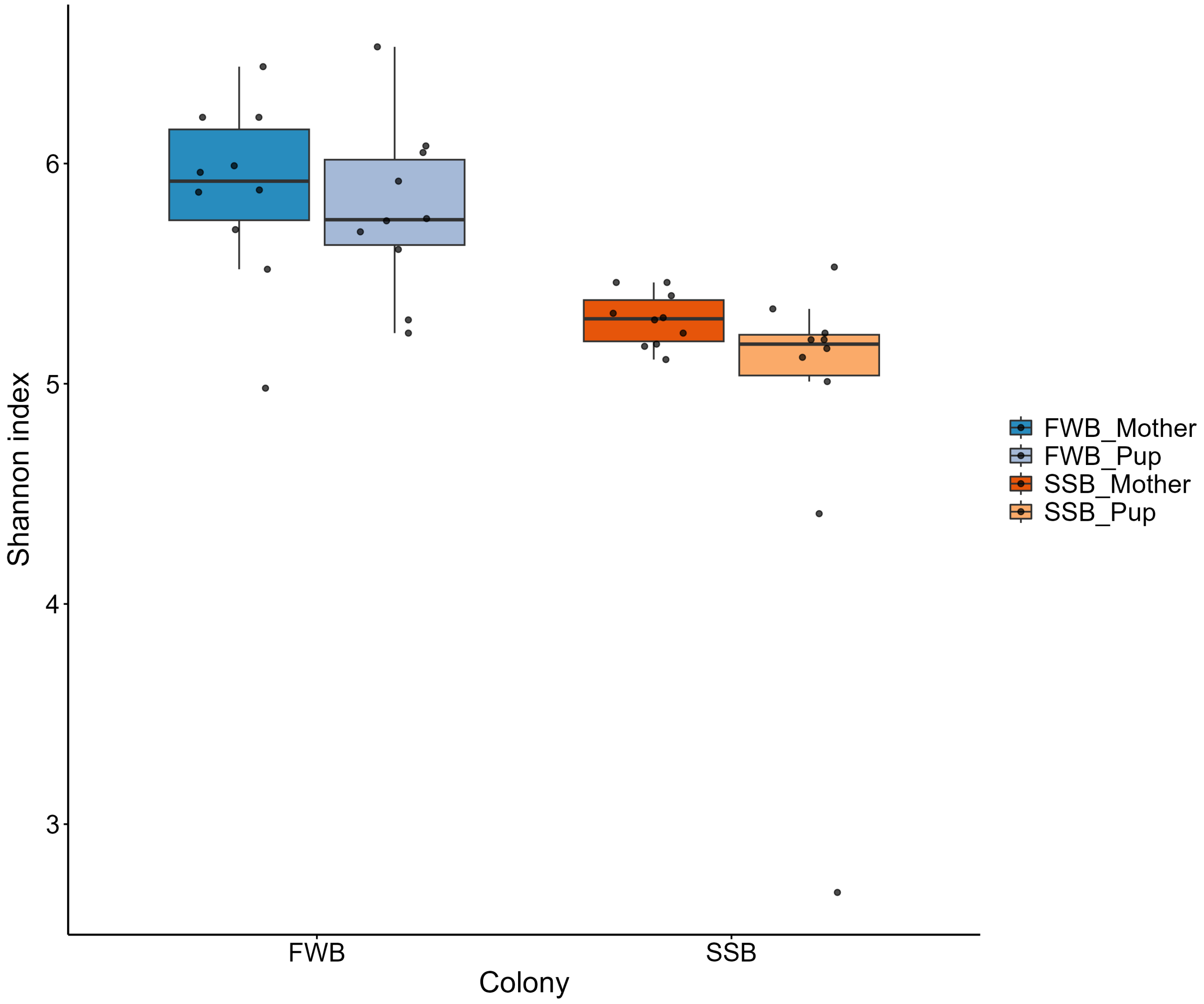

Supplementary tables

Table S1.

Sample IDs together with their corresponding metadata.

Sample_ID	Body_site	Colony	Age	ID_Pair	Sex	Weight_kg	Length_cm	Span_cm	Girth_cm
C1	Skin	FWB	Pup	F1	M	6.6	69.00	66.0	42.0
C1	Gut	FWB	Pup	F1	M	6.6	69.00	66.0	42.0
C17	Skin	FWB	Pup	F17	M	5.5	64.00	62.0	41.0
C17	Gut	FWB	Pup	F17	M	5.5	64.00	62.0	41.0
C18	Skin	FWB	Pup	F18	F	5.2	64.00	60.0	41.0
C18	Gut	FWB	Pup	F18	F	5.2	64.00	60.0	41.0
C21	Skin	FWB	Pup	F21	M	6.1	68.00	66.0	65.5
C21	Gut	FWB	Pup	F21	M	6.1	68.00	66.0	65.5

Sample_ID	Body_site	Colony	Age	ID_Pair	Sex	Weight_kg	Length_cm	Span_cm	Girth_cm
C22	Skin	FWB	Pup	F22	M	5.1	65.00	66.0	40.0
C22	Gut	FWB	Pup	F22	M	5.1	65.00	66.0	40.0
C25	Skin	FWB	Pup	F25	F	4.4	63.50	62.0	35.0
C25	Gut	FWB	Pup	F25	F	4.4	63.50	62.0	35.0
C3	Skin	FWB	Pup	F3	F	4.3	59.00	60.5	38.0
C3	Gut	FWB	Pup	F3	F	4.3	59.00	60.5	38.0
C5	Skin	FWB	Pup	F5	M	5.1	65.00	60.0	42.5
C5	Gut	FWB	Pup	F5	M	5.1	65.00	60.0	42.5
C6	Skin	FWB	Pup	F6	F	4.7	57.50	61.0	42.5
C6	Gut	FWB	Pup	F6	F	4.7	57.50	61.0	42.5
C7	Skin	FWB	Pup	F7	M	5.4	63.00	60.0	42.0
C7	Gut	FWB	Pup	F7	M	5.4	63.00	60.0	42.0
F1	Skin	FWB	Mother	C1	F	32.0	125.00	104.0	81.5
F1	Gut	FWB	Mother	C1	F	32.0	125.00	104.0	81.5
F17	Skin	FWB	Mother	C17	F	34.4	126.00	114.0	79.0
F17	Gut	FWB	Mother	C17	F	34.4	126.00	114.0	79.0
F18	Skin	FWB	Mother	C18	F	35.7	131.50	112.0	79.0
F18	Gut	FWB	Mother	C18	F	35.7	131.50	112.0	79.0
F21	Skin	FWB	Mother	C21	F	33.0	127.00	113.0	79.0
F21	Gut	FWB	Mother	C21	F	33.0	127.00	113.0	79.0
F22	Skin	FWB	Mother	C22	F	35.0	119.00	121.0	81.0
F22	Gut	FWB	Mother	C22	F	35.0	119.00	121.0	81.0
F25	Skin	FWB	Mother	C25	F	35.7	123.00	116.0	80.0
F25	Gut	FWB	Mother	C25	F	35.7	123.00	116.0	80.0
F3	Skin	FWB	Mother	C3	F	28.7	118.50	110.0	79.0
F3	Gut	FWB	Mother	C3	F	28.7	118.50	110.0	79.0
F5	Skin	FWB	Mother	C5	F	30.3	126.00	107.0	89.0
F5	Gut	FWB	Mother	C5	F	30.3	126.00	107.0	89.0
F6	Skin	FWB	Mother	C6	F	29.8	115.00	105.0	77.0
F6	Gut	FWB	Mother	C6	F	29.8	115.00	105.0	77.0
F7	Skin	FWB	Mother	C7	F	34.4	123.00	108.0	87.0
F7	Gut	FWB	Mother	C7	F	34.4	123.00	108.0	87.0
H11	Skin	SSB	Pup	S12	M	6.1	62.50	62.0	45.0
H11	Gut	SSB	Pup	S12	M	6.1	62.50	62.0	45.0
H13	Skin	SSB	Pup	S14	M	6.2	72.00	67.0	42.0
H13	Gut	SSB	Pup	S14	M	6.2	72.00	67.0	42.0
H15	Skin	SSB	Pup	S16	F	4.3	59.00	54.0	41.0
H15	Gut	SSB	Pup	S16	F	4.3	59.00	54.0	41.0
H16	Skin	SSB	Pup	S17	F	4.2	59.00	56.0	41.0
H16	Gut	SSB	Pup	S17	F	4.2	59.00	56.0	41.0
H18	Skin	SSB	Pup	S19	M	5.6	64.00	60.0	44.0
H18	Gut	SSB	Pup	S19	M	5.6	64.00	60.0	44.0
H19	Skin	SSB	Pup	S20	F	4.7	65.00	61.0	43.0
H19	Gut	SSB	Pup	S20	F	4.7	65.00	61.0	43.0
H2	Skin	SSB	Pup	S5	F	5.1	61.00	66.0	46.0
H2	Gut	SSB	Pup	S5	F	5.1	61.00	66.0	46.0
H20	Skin	SSB	Pup	S21	M	6.7	70.50	70.0	48.5
H20	Gut	SSB	Pup	S21	M	6.7	70.50	70.0	48.5
H21	Skin	SSB	Pup	S22	F	4.0	58.50	57.0	42.0
H21	Gut	SSB	Pup	S22	F	4.0	58.50	57.0	42.0
H22	Skin	SSB	Pup	S23	F	6.0	66.00	66.0	46.0
H22	Gut	SSB	Pup	S23	F	6.0	66.00	66.0	46.0
S12	Skin	SSB	Mother	H11	F	36.3	127.50	116.0	84.0
S12	Gut	SSB	Mother	H11	F	36.3	127.50	116.0	84.0
S14	Skin	SSB	Mother	H13	F	37.1	125.00	118.0	82.5

Sample_ID	Body_site	Colony	Age	ID_Pair	Sex	Weight_kg	Length_cm	Span_cm	Girth_cm
S14	Gut	SSB	Mother	H13	F	37.1	125.00	118.0	82.5
S16	Skin	SSB	Mother	H15	F	27.3	113.50	104.0	82.0
S16	Gut	SSB	Mother	H15	F	27.3	113.50	104.0	82.0
S17	Skin	SSB	Mother	H16	F	27.7	109.25	104.0	77.5
S17	Gut	SSB	Mother	H16	F	27.7	109.25	104.0	77.5
S19	Skin	SSB	Mother	H18	F	41.5	126.50	119.0	87.0
S19	Gut	SSB	Mother	H18	F	41.5	126.50	119.0	87.0
S20	Skin	SSB	Mother	H19	F	41.2	130.50	129.0	87.0
S20	Gut	SSB	Mother	H19	F	41.2	130.50	129.0	87.0
S21	Skin	SSB	Mother	H20	F	34.0	123.50	117.0	82.0
S21	Gut	SSB	Mother	H20	F	34.0	123.50	117.0	82.0
S22	Skin	SSB	Mother	H21	F	24.3	105.50	102.0	72.0
S22	Gut	SSB	Mother	H21	F	24.3	105.50	102.0	72.0
S23	Skin	SSB	Mother	H22	F	33.2	120.00	116.0	78.0
S23	Gut	SSB	Mother	H22	F	33.2	120.00	116.0	78.0
S5	Skin	SSB	Mother	H2	F	35.8	122.50	121.0	87.5
S5	Gut	SSB	Mother	H2	F	35.8	122.50	121.0	87.5
FWB soil	Control	NA	NA	NA	NA	NA	NA	NA	NA
SSB soil	Control	NA	NA	NA	NA	NA	NA	NA	NA
FWB air	Control	NA	NA	NA	NA	NA	NA	NA	NA
SSB air	Control	NA	NA	NA	NA	NA	NA	NA	NA
Gloves	Control	NA	NA	NA	NA	NA	NA	NA	NA
Hands	Control	NA	NA	NA	NA	NA	NA	NA	NA

Table S2.

Summary of total reads and ASVs for each sample before and after filtering steps.

Sample_ID	Reads_before	ASVs_before	Reads_after	ASVs_after	Colony	Age	Pair_ID	Sex
**Skin**								
S1	137872	2548	102058	1675	FWB	Mother	P1	F
S2	139601	2296	127035	1658	FWB	Mother	P2	F
S3	102354	1700	84223	1187	FWB	Mother	P3	F
S4	142368	1889	120277	1231	FWB	Mother	P4	F
S5	133357	1962	128985	1523	FWB	Mother	P5	F
S6	139142	2446	106173	1566	FWB	Mother	P6	F
S7	136650	2481	119116	1688	FWB	Mother	P7	F
S8	138275	2892	109980	1920	FWB	Mother	P8	F
S9	137085	2462	121119	1761	FWB	Mother	P9	F
S10	138423	2591	127313	1906	FWB	Mother	P10	F
S11	130377	2684	122930	2112	FWB	Pup	P1	M
S12	123451	2675	116922	1964	FWB	Pup	P2	M
S13	134272	2744	122955	1986	FWB	Pup	P3	F
S14	113392	1984	111418	1713	FWB	Pup	P4	M
S15	124015	2004	122480	1772	FWB	Pup	P5	M
S16	129911	2598	124469	2122	FWB	Pup	P6	F
S17	135878	2289	128200	1781	FWB	Pup	P7	F
S18	132064	2912	112272	2161	FWB	Pup	P8	M
S19	119875	2484	115733	2036	FWB	Pup	P9	F
S20	85319	2475	81363	1884	FWB	Pup	P10	M
S41	95783	1359	79198	1022	SSB	Mother	P11	F
S42	115688	1562	69159	1006	SSB	Mother	P12	F
S43	108110	1422	94681	1059	SSB	Mother	P13	F

Sample_ID	Reads_before	ASVs_before	Reads_after	ASVs_after	Colony	Age	Pair_ID	Sex
S44	106722	2221	104037	1806	SSB	Mother	P14	F
S45	139973	1835	130412	1257	SSB	Mother	P15	F
S46	137719	2330	118673	1478	SSB	Mother	P16	F
S47	132514	1883	122940	1492	SSB	Mother	P17	F
S48	138623	2006	133725	1539	SSB	Mother	P18	F
S49	140594	2191	129234	1530	SSB	Mother	P19	F
S50	102971	3358	53257	1357	SSB	Mother	P20	F
S51	128440	1254	118420	708	SSB	Pup	P11	M
S52	101788	1926	99039	1511	SSB	Pup	P12	M
S53	121989	2480	117808	2011	SSB	Pup	P13	F
S54	161803	2469	157676	1993	SSB	Pup	P14	F
S55	137203	2227	132252	1726	SSB	Pup	P15	M
S56	133413	2120	130611	1764	SSB	Pup	P16	F
S57	138054	1879	116671	1259	SSB	Pup	P17	F
S58	115139	1984	112402	1667	SSB	Pup	P18	M
S59	102756	2942	96133	1791	SSB	Pup	P19	F
S60	138753	1892	125142	1327	SSB	Pup	P20	F
**Gut**								
S21	128133	802	123735	693	FWB	Mother	P1	F
S22	121795	1007	119253	857	FWB	Mother	P2	F
S23	135288	604	130053	501	FWB	Mother	P3	F
S24	138047	1012	136810	812	FWB	Mother	P4	F
S25	133107	678	128822	558	FWB	Mother	P5	F
S26	114083	1454	111551	1097	FWB	Mother	P6	F
S27	134726	781	131082	606	FWB	Mother	P7	F
S28	132666	1027	131237	844	FWB	Mother	P8	F
S29	131133	895	119005	747	FWB	Mother	P9	F
S30	123419	639	115566	549	FWB	Mother	P10	F
S31	126797	1032	125141	798	FWB	Pup	P1	M
S32	130674	853	128000	708	FWB	Pup	P2	M
S33	135348	763	134367	598	FWB	Pup	P3	F
S34	131071	1689	123160	1208	FWB	Pup	P4	M
S35	123697	729	117175	548	FWB	Pup	P5	M
S36	130421	2324	118851	1490	FWB	Pup	P6	F
S37	129274	1054	127912	829	FWB	Pup	P7	F
S38	126113	1387	120236	1063	FWB	Pup	P8	M
S39	132930	1022	130513	736	FWB	Pup	P9	F
S40	125826	297	123578	261	FWB	Pup	P10	M
S61	115927	716	114144	594	SSB	Mother	P11	F
S62	119924	442	115951	384	SSB	Mother	P12	F
S63	138493	457	138133	393	SSB	Mother	P13	F
S64	139148	780	134705	609	SSB	Mother	P14	F
S65	132141	532	131508	451	SSB	Mother	P15	F
S66	117274	708	115771	556	SSB	Mother	P16	F
S67	115728	517	113255	422	SSB	Mother	P17	F
S68	128483	577	126712	497	SSB	Mother	P18	F
S69	121406	780	118184	590	SSB	Mother	P19	F
S70	129922	936	127375	733	SSB	Mother	P20	F
S71	136512	2378	114932	1390	SSB	Pup	P11	M
S72	124953	793	124043	622	SSB	Pup	P12	M
S73	133403	298	126803	218	SSB	Pup	P13	F
S74	124463	1315	115110	848	SSB	Pup	P14	F
S75	143075	1110	131069	666	SSB	Pup	P15	M
S76	129303	627	125755	512	SSB	Pup	P16	F
S77	103914	1495	68707	636	SSB	Pup	P17	F

Sample_ID	Reads_before	ASVs_before	Reads_after	ASVs_after	Colony	Age	Pair_ID	Sex
S78	132013	800	126028	677	SSB	Pup	P18	M
S79	134140	718	128959	609	SSB	Pup	P19	F
S80	133087	605	130486	520	SSB	Pup	P20	F

Table S3.

Total reads and ASVs for each body site (skin versus gut) before filtering steps.

Type	Total_reads	Mean_reads	SD_reads	Min_reads	Max_reads	Total_ASVs	Mean_ASVs	SD_ASVs	Min_ASVs	Max_ASVs
Skin & Gut	10209573	127619.7	12576.77	85319	161803	33624	1576.11	805.77	297	3358
Skin	5071716	126792.9	16029.29	85319	161803	27720	2236.40	460.30	1254	3358
Gut	5137857	128446.4	7878.19	103914	143075	11674	915.82	457.13	297	2378

Table S4.

Total reads and ASVs for each body site (skin versus gut) after filtering steps.

Type	Total_reads	Mean_reads	SD_reads	Min_reads	Max_reads	Total_ASVs	Mean_ASVs	SD_ASVs	Min_ASVs	Max_ASVs
Skin & Gut	9470168	118377.1	16595.84	53257	157676	7336	1154.74	563.14	218	2161
Skin	4546491	113662.3	19626.96	53257	157676	6946	1623.72	345.00	708	2161
Gut	4923677	123091.9	11273.38	68707	138133	3719	685.75	268.67	218	1490

Table S5.

Total counts for each phylum for each body site (skin versus gut).

Phylum	Reads	ASVs	Relative abundance (%)
**Skin**			
Pseudomonadota	1368589	2012	30.10
Bacillota	1181753	1144	25.99
Actinomycetota	787454	898	17.32
Bacteroidota	664958	2052	14.63
Fusobacteriota	362651	53	7.98
Patescibacteria	35242	88	0.78
Deinococcota	22539	31	0.50
Campylobacterota	21652	36	0.48
Chloroflexota	18100	85	0.40
Acidobacteriota	17419	150	0.38
Gemmatimonadota	12992	78	0.29
Thermodesulfobacteriota	12559	32	0.28
Cyanobacteria	11953	35	0.26
Deferribacterota	7354	10	0.16
Verrucomicrobiota	6723	75	0.15
Planctomycetota	4359	30	0.10
Myxococcota	4170	61	0.09
Bdellovibrionota	2217	33	0.05
Halobacterota	844	3	0.02
Nitrospirota	710	11	0.02
Abditibacteriota	457	6	0.01
Dependentiae	411	2	0.01

Phylum	Reads	ASVs	Relative abundance (%)
Spirochaetota	592	6	0.01
Synergistota	425	5	0.01
Armatimonadota	14	1	0.00
Caldisericota	29	1	0.00
Cloacimonadota	38	2	0.00
Fibrobacterota	81	3	0.00
Latescibacterota	10	1	0.00
Sumerlaeota	196	2	0.00
**Gut**			
Bacillota	1783757	780	36.23
Pseudomonadota	902317	1074	18.33
Bacteroidota	786854	1027	15.98
Fusobacteriota	767847	38	15.59
Actinomycetota	405457	495	8.23
Campylobacterota	251746	28	5.11
Thermodesulfobacteriota	6157	15	0.13
Patescibacteria	4920	54	0.10
Deferribacterota	4311	7	0.09
Acidobacteriota	3029	61	0.06
Deinococcota	2170	18	0.04
Cyanobacteria	1353	16	0.03
Verrucomicrobiota	1245	19	0.03
Gemmatimonadota	1164	23	0.02
Chloroflexota	637	30	0.01
Abditibacteriota	52	1	0.00
Bdellovibrionota	203	5	0.00
Cloacimonadota	6	1	0.00
Halobacterota	29	1	0.00
Myxococcota	205	11	0.00
Nitrospirota	52	4	0.00
Planctomycetota	75	4	0.00
Spirochaetota	14	3	0.00
Sumerlaeota	17	2	0.00
Synergistota	60	2	0.00

Table S6.

Core skin microbiota calculated as the shared ASVs among 90% of the individuals.

ASV	Phylum	Family	Genus	Species	Prevalence	Reads	Relative abundance (%)
ASV_1	Fusobacteriota	Fusobacteriaceae	Fusobacterium	mortiferum	40	136109	5.96
ASV_2	Fusobacteriota	Fusobacteriaceae	Fusobacterium	perfoetens	40	97930	4.29
ASV_3	Pseudomonadota	Moraxellaceae	Psychrobacter	NA	40	59742	2.61
ASV_4	Bacillota	Clostridiaceae	Clostridium sensu stricto 2	NA	40	101402	4.44
ASV_5	Pseudomonadota	Enterobacteriaceae	Escherichia-Shigella	coli	40	37876	1.66
ASV_6	Fusobacteriota	Leptotrichiaceae	Oceanivirga	NA	40	5316	0.23
ASV_7	Actinomycetota	Coriobacteriaceae	Collinsella	stercoris	40	77603	3.40
ASV_8	Bacillota	Peptostreptococcaceae	Peptoclostridium	NA	40	69971	3.06
ASV_10	Pseudomonadota	Moraxellaceae	Psychrobacter	NA	40	110080	4.82

ASV	Phylum	Family	Genus	Species	Prevalence	Reads	Relative abundance (%)
ASV_12	Bacillota	Clostridiaceae	Clostridium sensu stricto 2	NA	40	63041	2.76
ASV_13	Pseudomonadota	Moraxellaceae	Psychrobacter	urativorans	40	97401	4.26
ASV_14	Bacillota	Ruminococcaceae	Paludicola	NA	40	49739	2.18
ASV_16	Fusobacteriota	Fusobacteriaceae	Fusobacterium	NA	40	37404	1.64
ASV_17	Bacillota	Butyricicoccaceae	NA	NA	40	51276	2.24
ASV_19	Pseudomonadota	Moraxellaceae	Psychrobacter	arcticus	40	64623	2.83
ASV_20	Bacillota	Lachnospiraceae	[Eubacterium] fissicatena group	NA	40	35946	1.57
ASV_27	Bacillota	Lachnospiraceae	Lachnoclostridium	NA	40	32777	1.43
ASV_29	Actinomycetota	Micrococcaceae	Paeniglutamicibacter	NA	40	55043	2.41
ASV_30	Pseudomonadota	Moraxellaceae	Psychrobacter	NA	40	44080	1.93
ASV_33	Bacteroidota	Bacteroidaceae	Bacteroides	NA	40	19876	0.87
ASV_36	Bacillota	Lachnospiraceae	[Ruminococcus] gnavus group	NA	40	14768	0.65
ASV_37	Bacillota	Lachnospiraceae	Blautia	NA	40	34333	1.50
ASV_38	Pseudomonadota	Moraxellaceae	Psychrobacter	maritimus	40	33904	1.48
ASV_40	Actinomycetota	Intrasporangiaceae	Knoellia	NA	40	42241	1.85
ASV_41	Actinomycetota	Micrococcaceae	Arthrobacter	psychrochitiniphilus	40	39053	1.71
ASV_46	Bacillota	Peptostreptococcaceae	NA	NA	40	28782	1.26
ASV_48	Bacteroidota	Bacteroidaceae	Bacteroides	NA	40	8779	0.38
ASV_51	Bacillota	Lachnospiraceae	Blautia	hansenii	40	16597	0.73
ASV_52	Pseudomonadota	Moraxellaceae	Psychrobacter	cryohalolentis	40	23313	1.02
ASV_53	Bacillota	Family XI	Peptoniphilus	methioninivorax	40	1735	0.08
ASV_54	Bacillota	Clostridiaceae	Clostridium sensu stricto 1	perfringens	40	22164	0.97
ASV_55	Actinomycetota	Intrasporangiaceae	Intrasporangium	NA	40	30156	1.32
ASV_56	Bacillota	[Eubacterium] coprostanoligenes group	NA	NA	40	11225	0.49
ASV_62	Pseudomonadota	Moraxellaceae	Psychrobacter	cryohalolentis	40	20518	0.90
ASV_66	Bacillota	Clostridiaceae	Clostridium sensu stricto 1	perfringens	40	13968	0.61
ASV_67	Bacillota	Lachnospiraceae	Tuzzerella	NA	40	9163	0.40
ASV_75	Bacillota	Oscillospiraceae	UCG-005	NA	40	8609	0.38
ASV_76	Pseudomonadota	Pasteurellaceae	Otariodibacter	NA	40	6068	0.27
ASV_94	Pseudomonadota	Pasteurellaceae	Otariodibacter	NA	40	12951	0.57
ASV_101	Pseudomonadota	Moraxellaceae	Acinetobacter	NA	40	13064	0.57
ASV_102	Bacteroidota	Weeksellaceae	Ornithobacterium	NA	40	12764	0.56
ASV_116	Pseudomonadota	Comamonadaceae	Polaromonas	NA	40	11279	0.49
ASV_119	Deinococcota	Deinococcaceae	Deinococcus	marmoris	40	12058	0.53
ASV_121	Pseudomonadota	Neisseriaceae	Neisseria	zalophi	40	12828	0.56
ASV_122	Campylobacterota	Helicobacteraceae	Helicobacter	typhlonius	40	10983	0.48
ASV_141	Bacillota	Family XI	Anaerococcus	NA	40	10327	0.45
ASV_145	Thermodesulfobacteriota	Desulfovibrionaceae	NA	NA	40	8153	0.36
ASV_157	Pseudomonadota	Moraxellaceae	Psychrobacter	NA	40	9708	0.42
ASV_168	Pseudomonadota	Moraxellaceae	Psychrobacter	NA	40	8166	0.36
ASV_170	Bacillota	Ruminococcaceae	Fournierella	NA	40	3142	0.14
ASV_184	Pseudomonadota	Rhodanobacteraceae	Dokdonella	NA	40	6794	0.30
ASV_185	Deferribacterota	Deferribacteraceae	Mucispirillum	schaedleri	40	7046	0.31
ASV_229	Pseudomonadota	Enterobacteriaceae	Enterobacter	hormaechei	40	3174	0.14
ASV_236	Pseudomonadota	Moraxellaceae	Acinetobacter	baumannii	40	3693	0.16
ASV_241	Actinomycetota	Nocardiaceae	Rhodococcus	yunnanensis	40	5276	0.23

ASV	Phylum	Family	Genus	Species	Prevalence	Reads	Relative abundance (%)
ASV_242	Pseudomonadota	Moraxellaceae	Psychrobacter	NA	40	4278	0.19
ASV_260	Bacillota	Family XI	Tissierella	NA	40	4232	0.19
ASV_318	Bacillota	Peptostreptococcaceae	Romboutsia	NA	40	2940	0.13
ASV_346	Pseudomonadota	Rhodanobacteraceae	Rhodanobacter	NA	40	2984	0.13
ASV_516	Pseudomonadota	Alcaligenaceae	Achromobacter	NA	40	1372	0.06
ASV_11	Bacillota	Family XI	Helcococcus	NA	39	4890	0.21
ASV_18	Bacillota	Family XI	Ezakiella	massiliensis	39	3681	0.16
ASV_35	Bacillota	Family XI	Anaerococcus	NA	39	2484	0.11
ASV_39	Fusobacteriota	Fusobacteriaceae	Fusobacterium	mortiferum	39	18143	0.79
ASV_43	Bacteroidota	Bacteroidaceae	Bacteroides	NA	39	8628	0.38
ASV_49	Pseudomonadota	Moraxellaceae	Psychrobacter	NA	39	24184	1.06
ASV_58	Bacillota	Ruminococcaceae	Faecalibacterium	NA	39	3509	0.15
ASV_64	Bacillota	Oscillospiraceae	UCG-002	NA	39	6037	0.26
ASV_80	Bacteroidota	Bacteroidaceae	Bacteroides	NA	39	4179	0.18
ASV_81	Bacillota	Lachnospiraceae	Tyzzerella	NA	39	6365	0.28
ASV_83	Pseudomonadota	Moraxellaceae	Psychrobacter	cryohalolentis	39	14042	0.61
ASV_96	Bacillota	Clostridiaceae	Clostridium sensu stricto 1	NA	39	11566	0.51
ASV_108	Bacillota	Oscillospiraceae	UCG-005	NA	39	3160	0.14
ASV_109	Pseudomonadota	Moraxellaceae	Moraxella	NA	39	12830	0.56
ASV_118	Bacillota	Lactobacillaceae	Levilactobacillus	NA	39	12191	0.53
ASV_120	Actinomycetota	Micrococcaceae	Arthrobacter	agilis	39	11818	0.52
ASV_127	Actinomycetota	Dermacoccaceae	Allobranchiibius	NA	39	11953	0.52
ASV_134	Bacillota	Oscillospiraceae	UCG-005	NA	39	4873	0.21
ASV_147	Pseudomonadota	Moraxellaceae	Alkanindiges	NA	39	8848	0.39
ASV_179	Bacillota	Acidaminococcaceae	Phascolarctobacterium	NA	39	1926	0.08
ASV_182	Bacillota	Family XI	Parvimonas	NA	39	3341	0.15
ASV_218	Actinomycetota	Nocardioidaceae	Nocardioides	NA	39	6213	0.27
ASV_240	Actinomycetota	Dermacoccaceae	Allobranchiibius	NA	39	5668	0.25
ASV_262	Actinomycetota	Nakamurellaceae	Nakamurella	panacisegetis	39	4656	0.20
ASV_265	Bacillota	Family XI	Finegoldia	magna	39	3701	0.16
ASV_274	Actinomycetota	Micrococcaceae	Arthrobacter	psychrochitiniphilus	39	4360	0.19
ASV_289	Pseudomonadota	Moraxellaceae	Psychrobacter	NA	39	4273	0.19
ASV_310	Actinomycetota	Propionibacteriaceae	Tessaracoccus	NA	39	3576	0.16
ASV_331	Bacteroidota	Weeksellaceae	Ornithobacterium	NA	39	2713	0.12
ASV_23	Bacillota	Aerococcaceae	Atopobacter	phocae	38	6841	0.30
ASV_28	Bacteroidota	Bacteroidaceae	Bacteroides	NA	38	19297	0.84
ASV_32	Pseudomonadota	Moraxellaceae	Psychrobacter	urativorans	38	49008	2.15
ASV_44	Bacillota	Family XI	W5053	NA	38	1376	0.06
ASV_45	Pseudomonadota	Moraxellaceae	Psychrobacter	NA	38	38417	1.68
ASV_144	Bacillota	Oscillospiraceae	NK4A214 group	NA	38	1682	0.07
ASV_166	Actinomycetota	Intrasporangiaceae	Knoellia	NA	38	8947	0.39
ASV_176	Actinomycetota	Intrasporangiaceae	NA	NA	38	8194	0.36
ASV_200	Actinomycetota	Kineosporiaceae	Quadrisphaera	NA	38	6665	0.29
ASV_211	Bacillota	Clostridiaceae	Clostridium sensu stricto 1	paraputrificum	38	4963	0.22
ASV_215	Bacteroidota	Flavobacteriaceae	Flavobacterium	antarcticum	38	5407	0.24
ASV_228	Pseudomonadota	Moraxellaceae	Psychrobacter	NA	38	4337	0.19
ASV_231	Actinomycetota	Nocardiaceae	Nocardia	NA	38	5674	0.25
ASV_251	Bacillota	Ruminococcaceae	Fournierella	NA	38	935	0.04
ASV_252	Bacillota	Streptococcaceae	Streptococcus	marimammalium	38	4511	0.20
ASV_322	Pseudomonadota	Moraxellaceae	Psychrobacter	NA	38	2332	0.10
ASV_350	Actinomycetota	Dermacoccaceae	Allobranchiibius	NA	38	3236	0.14

ASV	Phylum	Family	Genus	Species	Prevalence	Reads	Relative abundance (%)
ASV_413	Actinomycetota	Corynebacteriaceae	Corynebacterium	phocae	38	2567	0.11
ASV_444	Bacillota	Lachnospiraceae	NA	NA	38	1769	0.08
ASV_456	Pseudomonadota	Enterobacteriaceae	Klebsiella	pneumoniae	38	1221	0.05
ASV_468	Bacillota	Ruminococcaceae	Faecalibacterium	prausnitzii	38	1434	0.06
ASV_485	Bacillota	Peptostreptococcaceae	Romboutsia	ilealis	38	946	0.04
ASV_665	Actinomycetota	Nocardiaceae	Nocardia	NA	38	1212	0.05
ASV_853	Actinomycetota	Intrasporangiaceae	NA	NA	38	1016	0.04
ASV_1621	Pseudomonadota	Moraxellaceae	Psychrobacter	NA	38	424	0.02
ASV_24	Actinomycetota	Actinomycetaceae	Arcanobacterium	NA	37	4006	0.18
ASV_60	Bacillota	Aerococcaceae	Atopobacter	phocae	37	3162	0.14
ASV_72	Bacillota	Family XI	Peptoniphilus	NA	37	1131	0.05
ASV_78	Bacteroidota	Rikenellaceae	Alistipes	NA	37	4214	0.18
ASV_79	Bacillota	Planococcaceae	Sporosarcina	NA	37	19551	0.86
ASV_126	Actinomycetota	Micrococcaceae	Arthrobacter	alpinus	37	11735	0.51
ASV_150	Bacillota	Oscillospiraceae	UCG-005	NA	37	2408	0.11
ASV_165	Pseudomonadota	Pasteurellaceae	Otariodibacter	NA	37	8238	0.36
ASV_201	Bacillota	Peptostreptococcaceae	Terrisporobacter	NA	37	4906	0.21
ASV_203	Actinomycetota	Ilumatobacteraceae	Ilumatobacter	NA	37	6639	0.29
ASV_226	Actinomycetota	Geodermatophilaceae	Antricoccus	NA	37	5521	0.24
ASV_230	Bacillota	Ruminococcaceae	Faecalibacterium	prausnitzii	37	3522	0.15
ASV_268	Pseudomonadota	Moraxellaceae	Psychrobacter	NA	37	3892	0.17
ASV_303	Actinomycetota	Dermacoccaceae	Allobranchiibius	NA	37	4065	0.18
ASV_304	Cyanobacteria	Leptolyngbyaceae	NA	NA	37	3304	0.14
ASV_323	Actinomycetota	Demequinaceae	NA	NA	37	2798	0.12
ASV_438	Bacillota	Lachnospiraceae	Lachnospiraceae NK4A136 group	NA	37	2125	0.09
ASV_477	Actinomycetota	Iamiaceae	Iamia	NA	37	2194	0.10
ASV_713	Bacillota	Ruminococcaceae	Fournierella	NA	37	1170	0.05
ASV_140	Bacillota	Streptococcaceae	Streptococcus	marimammalium	37	9303	0.41
ASV_244	Bacteroidota	Flavobacteriaceae	Flavobacterium	phocarum	37	4787	0.21

Table S7.

Core gut microbiota calculated as the shared ASVs among 90% of the individuals.

ASV	Phylum	Family	Genus	Species	Prevalence	Reads	Relative abundance (%)
ASV_1	Fusobacteriota	Fusobacteriaceae	Fusobacterium	mortiferum	40	224354	8.24
ASV_2	Fusobacteriota	Fusobacteriaceae	Fusobacterium	perfoetens	40	216034	7.93
ASV_3	Pseudomonadota	Moraxellaceae	Psychrobacter	NA	40	219772	8.07
ASV_4	Bacillota	Clostridiaceae	Clostridium sensu stricto 2	NA	40	125286	4.60
ASV_5	Pseudomonadota	Enterobacteriaceae	Escherichia-Shigella	coli	40	137668	5.06
ASV_6	Fusobacteriota	Leptotrichiaceae	Oceanivirga	NA	40	163092	5.99
ASV_7	Actinomycetota	Coriobacteriaceae	Collinsella	stercoris	40	86743	3.19
ASV_8	Bacillota	Peptostreptococcaceae	Peptoclostridium	NA	40	83974	3.08
ASV_10	Pseudomonadota	Moraxellaceae	Psychrobacter	NA	40	23945	0.88
ASV_11	Bacillota	Family XI	Helcococcus	NA	40	136068	5.00
ASV_12	Bacillota	Clostridiaceae	Clostridium sensu stricto 2	NA	40	71694	2.63

ASV	Phylum	Family	Genus	Species	Prevalence	Reads	Relative abundance (%)
ASV_16	Fusobacteriota	Fusobacteriaceae	Fusobacterium	NA	40	70609	2.59
ASV_17	Bacillota	Butyricicoccaceae	NA	NA	40	48359	1.78
ASV_18	Bacillota	Family XI	Ezakiella	massiliensis	40	88551	3.25
ASV_20	Bacillota	Lachnospiraceae	[Eubacterium] fissicatena group	NA	40	47149	1.73
ASV_27	Bacillota	Lachnospiraceae	Lachnoclostridium	NA	40	29117	1.07
ASV_30	Pseudomonadota	Moraxellaceae	Psychrobacter	NA	40	11663	0.43
ASV_37	Bacillota	Lachnospiraceae	Blautia	NA	40	17343	0.64
ASV_38	Pseudomonadota	Moraxellaceae	Psychrobacter	maritimus	40	14037	0.52
ASV_39	Fusobacteriota	Fusobacteriaceae	Fusobacterium	mortiferum	40	29788	1.09
ASV_41	Actinomycetota	Micrococcaceae	Arthrobacter	psychrochitiniphilus	40	5289	0.19
ASV_51	Bacillota	Lachnospiraceae	Blautia	hansenii	40	17484	0.64
ASV_53	Bacillota	Family XI	Peptoniphilus	methioninivorax	40	32052	1.18
ASV_56	Bacillota	[Eubacterium] coprostanoligenes group	NA	NA	40	20122	0.74
ASV_66	Bacillota	Clostridiaceae	Clostridium sensu stricto 1	perfringens	40	10487	0.39
ASV_67	Bacillota	Lachnospiraceae	Tuzzerella	NA	40	16479	0.61
ASV_75	Bacillota	Oscillospiraceae	UCG-005	NA	40	12914	0.47
ASV_229	Pseudomonadota	Enterobacteriaceae	Enterobacter	hormaechei	40	2189	0.08
ASV_236	Pseudomonadota	Moraxellaceae	Acinetobacter	baumannii	40	1965	0.07
ASV_14	Bacillota	Ruminococcaceae	Paludicola	NA	39	82335	3.02
ASV_19	Pseudomonadota	Moraxellaceae	Psychrobacter	arcticus	39	16096	0.59
ASV_29	Actinomycetota	Micrococcaceae	Paeniglutamicibacter	NA	39	6161	0.23
ASV_33	Bacteroidota	Bacteroidaceae	Bacteroides	NA	39	38046	1.40
ASV_35	Bacillota	Family XI	Anaerococcus	NA	39	53953	1.98
ASV_36	Bacillota	Lachnospiraceae	[Ruminococcus] gnavus group	NA	39	36488	1.34
ASV_44	Bacillota	Family XI	W5053	NA	39	38582	1.42
ASV_46	Bacillota	Peptostreptococcaceae	NA	NA	39	10312	0.38
ASV_54	Bacillota	Clostridiaceae	Clostridium sensu stricto 1	perfringens	39	9359	0.34
ASV_70	Bacillota	Aerococcaceae	Atopobacter	phocae	39	21907	0.80
ASV_72	Bacillota	Family XI	Peptoniphilus	NA	39	22606	0.83
ASV_13	Pseudomonadota	Moraxellaceae	Psychrobacter	urativorans	38	23753	0.87
ASV_40	Actinomycetota	Intrasporangiaceae	Knoellia	NA	38	3466	0.13
ASV_49	Pseudomonadota	Moraxellaceae	Psychrobacter	NA	38	6574	0.24
ASV_58	Bacillota	Ruminococcaceae	Faecalibacterium	NA	38	27403	1.01
ASV_62	Pseudomonadota	Moraxellaceae	Psychrobacter	cryohalolentis	38	4897	0.18
ASV_64	Bacillota	Oscillospiraceae	UCG-002	NA	38	22208	0.82
ASV_68	Bacillota	Family XI	Peptoniphilus	NA	38	24112	0.89
ASV_81	Bacillota	Lachnospiraceae	Tyzzerella	NA	38	13712	0.50
ASV_108	Bacillota	Oscillospiraceae	UCG-005	NA	38	12993	0.48
ASV_116	Pseudomonadota	Comamonadaceae	Polaromonas	NA	38	2003	0.07
ASV_516	Pseudomonadota	Alcaligenaceae	Achromobacter	NA	38	676	0.02
ASV_22	Campylobacterota	Campylobacteraceae	Campylobacter	blaseri	37	72903	2.68
ASV_24	Actinomycetota	Actinomycetaceae	Arcanobacterium	NA	37	65696	2.41
ASV_28	Bacteroidota	Bacteroidaceae	Bacteroides	NA	37	42842	1.57
ASV_43	Bacteroidota	Bacteroidaceae	Bacteroides	NA	37	31897	1.17
ASV_61	Bacteroidota	Porphyromonadaceae	Porphyromonas	NA	37	29438	1.08
ASV_115	Actinomycetota	Actinomycetaceae	Actinomyces	neuii	37	13729	0.50
ASV_122	Campylobacterota	Helicobacteraceae	Helicobacter	typhlonius	37	2294	0.08
ASV_124	Bacillota	Family XI	NA	NA	37	11796	0.43

ASV	Phylum	Family	Genus	Species	Prevalence	Reads	Relative abundance (%)
ASV_170	Bacillota	Ruminococcaceae	Fournierella	NA	37	5907	0.22
ASV_185	Deferribacterota	Deferribacteraceae	Mucispirillum	schaedleri	37	1280	0.05
ASV_242	Pseudomonadota	Moraxellaceae	Psychrobacter	NA	37	1405	0.05
ASV_318	Bacillota	Peptostreptococcaceae	Romboutsia	NA	37	899	0.03
ASV_337	Pseudomonadota	Moraxellaceae	Enhydrobacter	NA	37	596	0.02
ASV_823	Bacillota	Clostridiaceae	Clostridium sensu stricto 1	NA	37	558	0.02

Table S8.

Output of the linear mixed effect models.

Shannon index	Estimate	SE	Df	CI_lower	CI_upper	Chisq	Pr(>F)
**Skin**							
Intercept	5.95	0.14	28.31	5.68	6.23	-	-
Colony (SSB)	-0.74	0.18	1	-1.08	-0.42	17.51	<0.001
Age (Pup)	-0.24	0.14	1	-0.52	0.04	3.08	0.08
**Gut**							
Intercept	3.52	0.21	32.5	3.09	3.93	-	-
Colony (SSB)	-0.07	0.25	1	-0.53	0.39	0.09	0.761
Age (Pup)	0.06	0.24	1	-0.43	0.58	0.07	0.792

Table S9.

Output of the linear models.

Shannon index	Estimate	SE	Df	CI_lower	CI_upper	F-value	P-value
**Skin-pups**							
Intercept	6.09	0.25	1	5.56	6.63	-	-
Colony (SSB)	-1	0.28	1	-1.58	-0.42	11.06	<0.001
Sex (Male)	-0.51	0.28	1	-1.09	0.08	3.36	0.08
**Gut-pups**							
Intercept	3.52	0.44	1	2.59	4.46	-	-
Colony (SSB)	0.07	0.48	1	-0.95	1.09	0.02	0.883
Sex (Male)	-0.02	0.48	1	-1.04	1.01	1e-03	0.975

Table S10.

Output of PERMANOVA analysis and dispersion test.

	Permanova					betadisper	
Parameter	Df	SumOfSqs	R2	F (P)	Pr(>F) (P)	F (b)	Pr(>F) (b)
**Skin**							
Colony	1	1.662	0.228	15.462	0.001	1.832	0.184
Age	1	0.292	0.04	2.716	0.007	11.132	0.002
Pair_ID	18	3.277	0.451	1.694	0.001	-	-
Residual	19	2.042	0.281	-	-	-	-
Total	39	7.272	1	-	-	-	-

	Permanova					betadisper	
Parameter	Df	SumOfSqs	R2	F (P)	Pr(>F) (P)	F (b)	Pr(>F) (b)
**Gut**							
Colony	1	0.601	0.081	3.829	0.001	0.334	0.567
Age	1	0.695	0.094	4.431	0.001	18.564	<0.001
Pair_ID	18	3.118	0.422	1.104	0.102	-	-
Residual	19	2.982	0.403	-	-	-	-
Total	39	7.396	1	-	-	-	-
**Skin-pups**							
Sex	1	0.139	0.046	1.12	0.232	2.129	0.162
Colony	1	0.803	0.263	6.466	0.001	0.033	0.858
Residual	17	2.11	0.691	-	-	-	-
Total	19	3.052	1	-	-	-	-
**Gut-pups**							
Sex	1	0.208	0.055	1.117	0.276	0.207	0.654
Colony	1	0.439	0.115	2.354	0.001	0.002	0.963
Residual	17	3.172	0.83	-	-	-	-
Total	19	3.82	1	-	-	-	-
